# Details of oral anticancer drug prescription audits by community pharmacists: a retrospective analysis of prescription inquiries

**DOI:** 10.1186/s40780-026-00580-4

**Published:** 2026-05-07

**Authors:** Yuma Shibutani, Keito Ikou, Shinya Suzuki, Sayaka Nakajima, Akiko Hashimoto, Azumi Sako, Naoko Kumazawa, Yasuaki Ryusima, Masahito Yonemura, Naoki Kondo

**Affiliations:** Department of Pharmacy, National Cancer Center Hospital East, 6-5-1 Kashiwanoha, Kashiwa, Chiba 277-8577 Japan

**Keywords:** Oral anticancer drugs, Community pharmacists, Prescription audit, Outpatient cancer pharmacotherapy, Hospital pharmacists

## Abstract

**Background:**

Cancer pharmacotherapy has shifted to outpatient settings, making community pharmacists essential for ensuring medication safety. Although community pharmacist prescription audits are important, evidence regarding the clinical significance of their prescription audits for oral anticancer agents remains limited. This study aimed to analyze the content of inquiries regarding oral anticancer drugs and clarify the details of prescription audits conducted by community pharmacists.

**Methods:**

This single-institution retrospective a descriptive observational study included all records of inquiries regarding oral anticancer drugs submitted from community pharmacies to the National Cancer Center Hospital East between September 2023 and March 2025. Inquiry content was categorized based on treatment efficacy and safety concerns, while the level of pharmacist intervention was assessed based on the severity of the medication error and the clinical value of the pharmacist service. As the purpose of this study is to investigate prescription audits by community pharmacies, interventions unrelated to prescription auditing, such as telephone follow-ups or tracing reports, were excluded.

**Results:**

During the study period, 184,688 prescriptions were issued, of which 384 inquiries (0.2%) involved oral anticancer agents and were included in the analysis. Prescription modifications occurred in 295 cases (77%). The most common inquiry categories were incorrect treatment duration (23%), adjustment for leftover medication (20%), and dosage errors (16%). Overall, 49% of cases were classified as potentially lethal, serious, or significant medication-order errors, and 49% were assessed as having a value of service of significant or higher. High-severity and high-value interventions most frequently involved errors in treatment duration and dosage.

**Conclusions:**

Prescription inquiries regarding oral anticancer agents frequently identified clinically significant prescribing issues, and community pharmacists provided high-value interventions. These findings indicate that community pharmacists play a crucial role in conducting high-quality prescription audits and enhancing the safety of outpatient cancer drug therapy.

**Supplementary Information:**

The online version contains supplementary material available at 10.1186/s40780-026-00580-4.

## Background

In recent years, with the increasing use of oral anticancer agents, cancer pharmacotherapy has been shifting from an inpatient-centered model to an outpatient-centered model [1–3]. Patients receiving anticancer agents may have complex medication requirements related to dosage, treatment schedules, and the management of adverse events [4–6]. Therefore, to enable patients to continue treatment safely at home, involvement in patient safety management is required not only in hospital pharmacies but also in community pharmacies [7].

In Japan, patients prescribed oral anticancer agents receive these medications at community pharmacies [8, 9]. Accordingly, community pharmacists provide patient management, including prescription verification, dispensing, patient counseling, and monitoring for potential adverse events [10]. When questions arise regarding a prescription, community pharmacists contact hospital physicians or pharmacists, and the medication is dispensed to the patient after the issue has been resolved. Thus, timely information sharing and close collaboration between community pharmacies and hospitals are essential [8].

Previous studies have reported that pharmacist involvement enables early detection of adverse events and modification of prescriptions during outpatient anticancer treatment [11–14]. However, reports on the involvement of community pharmacists in the care of patients undergoing outpatient chemotherapy remain limited. In particular, although prescription verification by community pharmacists is considered to play an important role in successful treatment, detailed investigations of their interventions are scarce, the evidence regarding community pharmacists’ ability to audit prescriptions for oral anticancer drugs remains unclear.

Therefore, we conducted a detailed investigation of the inquiry content to clarify the audit capabilities of community pharmacists regarding oral anticancer drug prescriptions.

## Methods

### Study design and setting

This was a single-center, retrospective a descriptive observational study aimed at clarifying the content of inquiries and the level of pharmacist interventions regarding prescriptions for oral anticancer drugs in community pharmacies. We included inquiries made by community pharmacies regarding outpatient prescriptions issued by the National Cancer Center Hospital East, Japan, between September 1, 2023, and March 31, 2025. This study was conducted in accordance with the Declaration of Helsinki and was approved by the Institutional Review Board of the National Cancer Center (Research No: 2025 − 140).

### Data source and data collection

At our hospital, more than 90% of outpatients receive their medications at community pharmacies based on prescriptions’ prescriptions, except for certain anticancer agents that must be dispensed by hospital pharmacists. Community pharmacists confirm prescription details by telephone with the hospital when they have questions about a prescription. When a hospital pharmacist received a call from a community pharmacy, the pharmacist obtained detailed information regarding the inquiry, categorized its content, consulted the prescribing physician as needed, and provided feedback to the community pharmacist. Hospital pharmacists documented in the electronic medical record the feedback provided to community pharmacies, the medications involved, whether the prescription was modified after contacting the prescribing physician, and, if modified, the reason for the change. In this study, we retrospectively extracted all records of inquiries regarding oral anticancer drugs during the study period and examined their content and the level of pharmacist interventions.

### Classification of inquiry content and intervention level

The content of inquiries from community pharmacies was categorized based on the framework described by Barnett et al. (2009) [15], with drug therapy problems classified from the perspectives of efficacy and safety (Supplementary Table 1). The level of pharmacist intervention was assessed using the rating instrument reported by Overhage and Lukes (1999) [16], and interventions were analyzed according to the severity of medication-order errors and the value of pharmacist clinical services (Supplementary Tables 2 and 3). The severity of medication-order errors was assessed based on the potential clinical impact that could have occurred if the error had not been identified. Specifically, errors were classified as potentially lethal, serious, significant, or minor according to the expected degree of harm to the patient. Errors related to treatment duration were evaluated based on their potential to cause clinically relevant consequences, such as overdose, inappropriate prolongation of therapy, or treatment interruption. Errors likely to result in these outcomes were classified as potentially lethal or serious, whereas those with limited clinical impact were classified as significant or minor. Although these two assessments represent distinct concepts, corresponding relationships were observed between the categories of severity (potentially lethal, serious, and significant) and the categories of value of service (extremely significant, very significant, and significant). Therefore, in this study, corresponding categories were used to ensure consistency in classification based on the same evaluation framework.

All evaluations were performed by pharmacists based on clinical information documented in the electronic medical records. When there was uncertainty in classification, the cases were reviewed by multiple pharmacists, and the final classification was determined through consensus.

### Study outcomes

The primary outcomes were the classification of the content of prescription audit inquiries from community pharmacies and the level of pharmacist interventions. Therefore, interventions and reports from community pharmacists related to telephone follow-up and tracing reports, which were not considered prescription auditing, were excluded. The primary objective of this study was to descriptively characterize community pharmacists’ prescription audits in the management of oral anticancer drugs, therefore no statistical analyses were performed.

## Results

### Number of cases included in the analysis

**Fig. 1:**
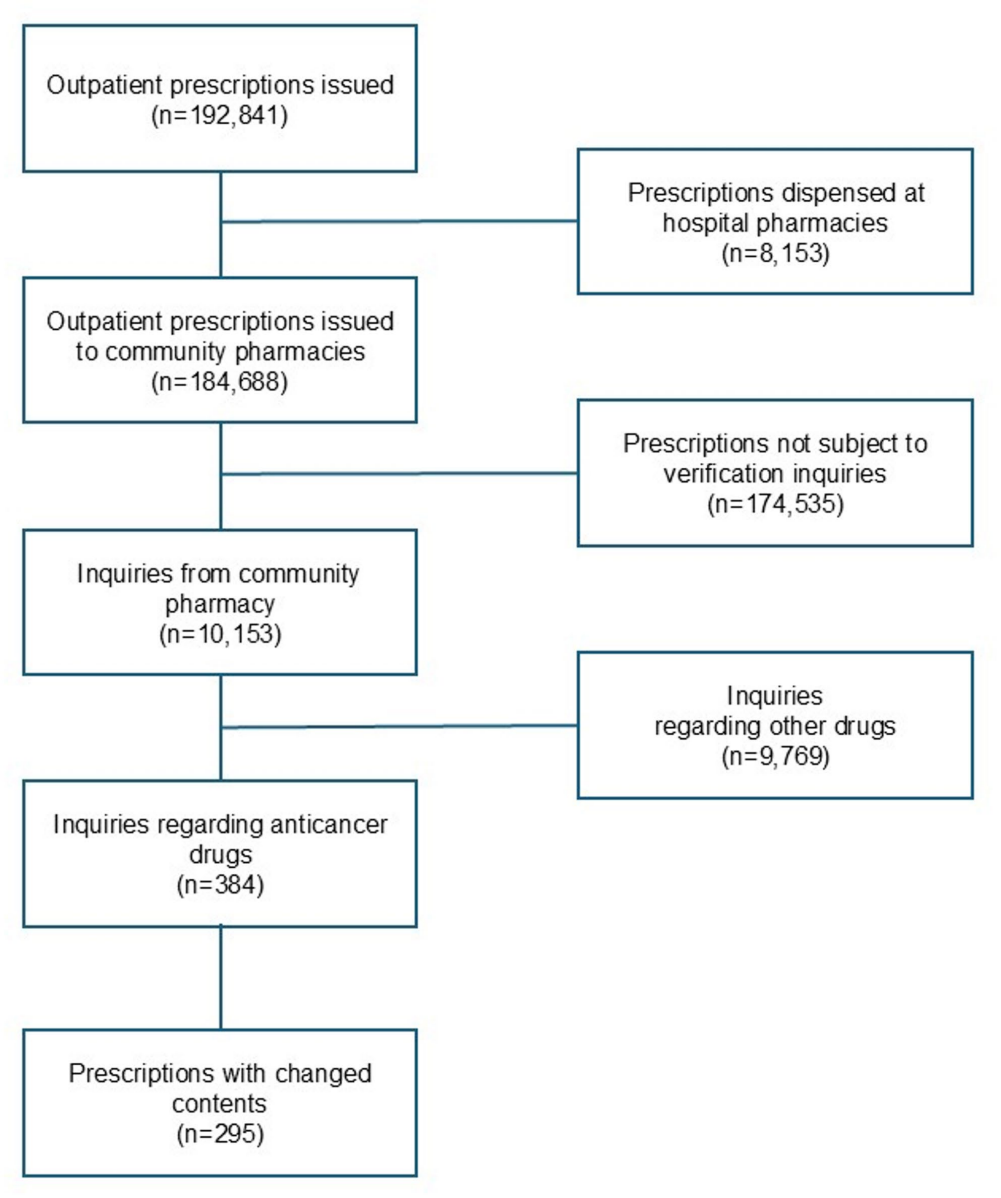
Flow diagram of extracted inquiry cases

The total number of prescriptions issued during the survey period was 192,841. Of these, 184,688 (96%) were dispensed at community pharmacies (Fig. 1). Among these, pharmacists at community pharmacies contacted hospitals regarding the prescription details for 10,153 prescriptions (5%). Of these cases, 384 were related to oral anticancer drugs, accounting for 0.2% of all prescriptions issued during the study period. These 384 cases were included for detailed analysis. Of the 384 prescriptions examined, 295 (77%) were modified. Prescription modification rates differed according to the level of pharmacist intervention. All cases classified as extremely significant, all 19 cases (100%) resulted in prescription changes. Among very significant cases, 48 of 62 cases (77%) were modified, while 68 of 113 significant cases (60%) resulted in prescription changes. Higher intervention levels were associated with higher prescription modification rates.

A list of anticancer drugs subject to inquiry is shown in Supplementary Table 4. The most frequently inquired anticancer drug was tegafur/gimeracil/oteracil potassium with 106 cases (28%), followed by capecitabine with 58 cases (15%), and trifluridine tipiracil with 37 cases (10%).

### Details of inquiry content

Among the 384 cases included in the analysis, 97 cases (25%) were related to drug therapy efficacy, primarily concerning the dosage or treatment duration of anticancer drugs (Table 1). In contrast, 112 cases (29%) were related to drug therapy safety, mainly concerning potential overdosing or treatment duration. The detailed classification of inquiry content is summarized in Table 2. The most frequent category was an incorrect treatment duration (87 cases, 23%), followed by adjustment for leftover medication (76 cases, 20%), dosage error (60 cases, 16%), and an incorrect start date of administration (34 cases, 9%).

**Table 1:** Details of inquiry content

	Indication for MTM Service (REASON)	Total number (384), (%)
Drug Therapy Efficacy	Suboptimal drug selection	0
	Insufficient dose or duration	97 (25.2)
Drug Therapy Safety	Adverse drug reaction	0
	Drug interaction	0
	Excessive dose or duration	112 (29.2)
Other		175 (45.6)

MTM = medication therapy management

**Table 2:** Detailed categories of inquiries related to oral anticancer drugs

Category	*n* (%)
Prescription duration error	87 (22.7)
Adjustment for leftover medication	76 (19.8)
Dosage error	60 (15.6)
Confirmation of the start date of treatment	34 (8.9)
Change of prescription medication at the patient’s request	33 (8.6)
Incorrect administration instructions	25 (6.5)
Omission of prescription	18 (4.7)
Out of stock / change in dosage form	15 (3.9)
Confirmation of treatment continuation	12 (3.1)
Patient request: switch to a lower-cost medication	7 (1.8)
Prescription errors involving drug names	5 (1.3)
Patient request / change in dosage form	4 (1.0)
Feasibility of one-dose packaging	1 (0.3)

### Level of intervention

For the 384 cases analyzed, we further classified and evaluated both the severity of medication-order errors and the value of pharmacist interventions. Regarding the severity of medication-order errors, 213 cases (55%) involved medication-order errors ranging from potentially lethal to minor, for which pharmacist interventions were provided (Table 3). In addition, 188 cases (49%) were classified as potentially lethal, serious, or significant medication-order errors. Regarding the value of pharmacist interventions, 19 cases (5%) were classified as extremely significant, 61 cases (16%) as very significant, and 108 cases (28%) as significant. Overall, 188 cases (49%) were classified as having a value of service of significant or higher.

**Table 3:** Details of intervention levels

Category	n (%)
**Severity level**	
Potentially lethal	19 (4.9)
Serious	62 (16.1)
Significant	106 (27.6)
Minor	25 (6.5)
No error	172 (44.8)
**Value of service**	
Extremely significant	19 (4.9)
Very significant	62 (16.1)
Significant	106 (27.6)
Somewhat significant	25 (6.5)
No significance	172 (44.8)
Adverse significance	0

When case content was examined by intervention level, the most common categories among cases classified as potentially lethal and extremely significant were incorrect treatment duration (8 cases), followed by dosage error (6 cases) and omission of a prescription (5 cases) (Table 4). Among cases classified as serious and very significant, the most frequent category was dosage error (25 cases), followed by incorrect treatment duration and incorrect administration instructions. Among cases classified as significant, the most frequent category was incorrect treatment duration (43 cases), followed by dosage error (23 cases).

**Table 4:** Details on severity and value of interventions

Detailed inquiry content	*n* (%)
Potentially lethal / Extremely significant (total number: 19)	
Prescription duration error	8 (42.1)
Dosage error	6 (31.6)
Prescription oversight	5 (26.3)
Serious / Very significant (total number: 62)	
Dosage error	25 (40.3)
Prescription duration error	15 (24.2)
Incorrect administration instructions	8 (12.9)
Prescription oversight	7 (11.3)
Confirmation of treatment continuation	4 (6.5)
Confirmation of the start date of treatment	3 (4.8)
Significant / Significant (total number: 106)	
Prescription duration error	43 (40.6)
Dosage error	23 (21.7)
Confirmation of the start date of treatment	17 (16.0)
Incorrect administration instructions	14 (13.2)
Confirmation of treatment continuation	6 (5.7)
Prescription oversight	3 (2.8)

The severity of medication-order errors was assessed based on the potential clinical impact on the patient if the error had not been corrected. The value of pharmacist interventions was assessed based on the clinical significance of the intervention in improving patient care and medication safety

## Discussion

In this single-center retrospective descriptive study, we conducted a detailed analysis of the content of inquiries made by community pharmacists to our hospital regarding oral anticancer drug prescriptions. To our knowledge, this is one of the first studies to characterize both the detailed content of prescription inquiries and the level of pharmacist interventions in this setting. Nearly half of the cases were classified as having high clinical significance, with 49% categorized as potentially lethal, serious, or significant medication-order errors and 49% assessed as having a value of service of significant or higher. Furthermore, the prescription change rate following inquiries from community pharmacists was 77%, suggesting that pharmacist-led prescription audits frequently lead to clinically relevant modifications. These findings suggest that community pharmacists may contribute to identifying clinically important prescribing issues in outpatient cancer pharmacotherapy.

The most frequent inquiry category was an incorrect treatment duration, followed by adjustment for leftover medication and dosage errors. For oral anticancer drug regimens such as tegafur/gimeracil/oteracil potassium and capecitabine may involve treatment schedules tailored to individual patients, and dosage adjustments frequently occur [17, 18]. Previous studies have reported that dosage-related errors are the most common type of intervention by community pharmacists, while errors related to treatment duration have also been identified as an important concern [19, 20]. Consistent with previous studies, a high number of inquiries in this study were related to errors in treatment duration and dosage, indicating a similar trend to earlier findings. As this study focused on oral anticancer agents, errors related to treatment duration may have been more frequently observed. Currently, the administration of oral anticancer drugs is becoming increasingly complex, and errors in treatment duration or dosage can lead to clinically significant consequences such as unintended overdose or treatment interruption. In particular, errors in treatment duration are the most frequent category even among the highest-risk patient groups requiring intervention, necessitating careful attention to ensure the safe administration of drug therapy to patients.

Adjustment for leftover medication was the second most common inquiry category in this study. This may be attributable to the real-world variability of outpatient cancer treatment, including changes in visit intervals, temporary treatment interruptions due to adverse events, dose modifications, or delays in treatment initiation. Such adjustments are particularly important for oral anticancer drugs, as inappropriate continuation or duplication of therapy may increase the risk of toxicity, while insufficient supply may lead to treatment interruption. Although hospital pharmacists can also perform these duties [19], the number of hospital pharmacists is limited, making it difficult to provide adequate patient management in some cases. Therefore, the role of community pharmacists in the management of patients with cancer has become increasingly important. The results of this study suggest that community pharmacists may contribute not only to error prevention but also to maintaining continuity and adherence in outpatient cancer care.

The assessment of the severity of medication-order errors and the value of pharmacist interventions in this study was based on established frameworks and clinical judgment. This approach reflects real-world clinical practice and has been widely used in the evaluation of medication errors. However, these assessments rely on evaluator judgment and may be subject to inter-rater variability and classification bias. In addition, both assessments were conducted using a shared classification framework, which may have contributed to the observed correspondence between severity and value. Furthermore, these evaluations were based on the potential clinical impact of errors rather than actual patient outcomes; therefore, the results should be interpreted with caution. Furthermore, we did not assess whether community pharmacists intervened in all clinically relevant issues; therefore, some problems may have been missed, which could have influenced the interpretation of the results.

Given the increasing shift toward outpatient-centered cancer pharmacotherapy, effective collaboration and timely information sharing between hospitals and community pharmacies are essential. Community pharmacists have been reported to face limitations in the amount of patient information available from prescriptions and to often feel that it is not easy to consult hospital pharmacists [21]. In contrast, our findings suggest that community pharmacists frequently detect errors in prescription duration and dosage and are often involved in clinically important issues that require prompt confirmation and correction. Therefore, to ensure the safe continuation of cancer pharmacotherapy, establishing closer and more effective communication between hospital pharmacists and community pharmacists is essential. Potential approaches to address these challenges include more clearly documenting the intended treatment duration and the start date of administration on prescriptions and strengthening communication tools that facilitate timely confirmation between hospital pharmacists and community pharmacists. In addition, collaborative efforts between hospitals and community pharmacies to establish prescription-checking systems may help reduce duration- and dosage-related errors.

This study has several limitations. First, this was a single-center retrospective study, and the generalizability of the findings may be limited. Second, this analysis is based on data recorded in electronic medical records, and cases recorded or managed by alternative means may have been overlooked. Third, Third, we did not assess the clinical experience of community or hospital pharmacists. Because the National Cancer Center Hospital East does not share the electronic medical record system with community pharmacies, pharmacists’ clinical experience may have influenced the quality of prescription auditing and introduced potential bias into the results. Finally, the assessment of severity and value of service relied on subjective judgment and may be subject to inter-rater variability, expectation bias, and misclassification. Future multicenter studies evaluating the clinical impact of community pharmacist interventions are warranted.

This study revealed that community pharmacists are conducting high-quality prescription audits. To further enhance patient care, strengthening collaboration between hospital pharmacists and community pharmacists is essential.

## Conclusions

This study found that inquiries regarding oral anticancer drug prescriptions frequently involved clinically significant issues and high-value pharmacist interventions. These findings suggest that community pharmacists may play an important role in enhancing the safety and quality of outpatient cancer pharmacotherapy through prescription verification and timely communication with hospitals.

## Supplementary Information

Below is the link to the electronic supplementary material.

Supplementary Material 1

## Data Availability

The data supporting the findings of this study are available from the corresponding author upon reasonable request.
