# Supplementary material for: Details of oral anticancer drug prescription audits by community pharmacists: a retrospective analysis of prescription inquiries
Source: J Pharm Health Care Sci. 2026 May 7;12:60. doi: 10.1186/s40780-026-00580-4 (PMC13321446; doi:10.1186/s40780-026-00580-4)
Supplement: Supplementary file 1 — Supplementary Material 1 [file 40780_2026_580_MOESM1_ESM.docx]

***Supplemental Tables and supporting information***

**Supplemental Table 1: MTM intervention classification: Drug therapy efficacy and safety categories**

|  | Indication for MTM Service (REASON) | Description/Examples |
| --- | --- | --- |
| Drug Therapy Efficacy | Suboptimal drug selection | An order to initiate or continue a drug therapy with suboptimal efficacy (e.g., patient with systolic heart failure receives a new prescription for propranolol or other beta-blocker not shown to decrease mortality). |
|  | Insufficient dose or duration | An order to initiate or continue drug therapy at a dose or duration insufficient to be effective (e.g., a patient presents with uncontrolled blood sugar and is not on optimal dose of antidiabetic medication). |
| Drug Therapy Safety | Adverse drug reaction | A drug order with an adverse reaction risk significant enough to render the therapy unsafe, including side effects and allergic or idiosyncratic reactions (e.g., patient is on statin therapy and reports leg pain). |
|  | Drug interaction | A drug order with a drug interaction risk significant enough to render the therapy unsafe (e.g., patient is prescribed sildenafil and a nitrate by different prescribers). |
|  | Excessive dose or duration | An order to initiate or continue drug therapy at a dose or duration too excessive to be safe (e.g. antibiotic for a 6 year-old patient prescribed at an adult dosage). |

MTM = medication therapy management

**Supplemental Table 2. Severity of error categories for medication orders**

| Severity level | Description / examples |
| --- | --- |
| Potentially lethal | High potential for life-threatening adverse reactions; potentially lifesaving drug at too low a dosage; extremely high dosage (>10× normal) of a drug with low therapeutic index |
| Serious | Route could lead to severe toxicity; dosage resulting in potentially toxic serum concentration; drug could exacerbate patient condition; documented allergy to drug; omission of hypersensitivity pretest |
| Significant | Dose too low for condition; high dosage (1.5–4× normal) of drug with low therapeutic index; errant dual-drug therapy; inappropriate dosing interval; omission from medication order |
| Minor | Incomplete information; unavailable/inappropriate dosage form; nonformulary drug; noncompliance with standard formulations/policies; illegible/ambiguous orders |
| No error | Information or clarification requested from pharmacist; cost savings only |

**Supplemental Table 3. Value of service categories for pharmacists’ clinical interventions**

| Value of service | Description |
| --- | --- |
| Extremely significant | Recommendation with extremely serious consequences or potential life-and-death situation |
| Very significant | Recommendation involving potential or existing dysfunction in a major organ; avoidance of serious adverse drug interaction or contraindication |
| Significant | Recommendation improves care to an acceptable/appropriate level (standard of practice), including quality-of-life issues, cost, and convenience |
| Somewhat significant | Patient benefit may be neutral depending on professional interpretation; clarification required before order can be processed |
| No significance | Information only; recommendation not patient-specific |
| Adverse significance | Recommendation inappropriate; implementation may lead to adverse outcomes |

**Supplemental Table 4. Anticancer drugs subject to inquiry**

| **Anticancer drug** | **Number of cases, (%)** |
| --- | --- |
| Tegafur / Gimeracil / Oteracil | 106 (27.6) |
| Capecitabine | 58 (15.1) |
| Trifluridine / Tipiracil | 37 (9.6) |
| Palbociclib | 20 (5.2) |
| Imatinib | 18 (4.7) |
| Niraparib | 17 (4.4) |
| Abiraterone | 16 (4.2) |
| Dabrafenib / Trametinib | 12 (3.1) |
| Abemaciclib | 12 (3.1) |
| Lenvatinib | 12 (3.1) |
| Tegafur / Uracil | 11 (2.9) |
| Regorafenib | 11 (2.9) |
| Osimertinib | 8 (2.1) |
| Trametinib | 6 (1.6) |
| Fruquintinib | 5 (1.3) |
| Bicalutamide | 4 (1.0) |
| Talazoparib | 3 (0.8) |
| Hydroxycarbamide | 3 (0.8) |
| Olaparib | 3 (0.8) |
| Cabozantinib | 3 (0.8) |
| Nilotinib | 2 (0.5) |
| Dabrafenib | 2 (0.5) |
| Darolutamide | 2 (0.5) |
| Alectinib | 1 (0.3) |
| Enzalutamide | 1 (0.3) |
| Pazopanib | 1 (0.3) |
| Acalabrutinib | 1 (0.3) |
| Afatinib | 1 (0.3) |
| Pirtobrutinib | 1 (0.3) |
| Pimitespib | 1 (0.3) |
| Tepotinib | 1 (0.3) |
| Temozolomide | 1 (0.3) |
| Capivasertib | 1 (0.3) |
| Futibatinib | 1 (0.3) |
| Encorafenib / Binimetinib | 1 (0.3) |
| Entrectinib | 1 (0.3) |
